# Blockade of caspase cascade overcomes malaria-associated acute respiratory distress syndrome in mice

**DOI:** 10.1038/s41419-022-04582-6

**Published:** 2022-02-10

**Authors:** Michelle K. Sercundes, Luana S. Ortolan, Viviane da Silva Julio, Leonardo M. Bella, Thatyane de Castro Quirino, Daniela Debone, Marcela S. Carneiro-Ramos, Marcelo A. Christoffolete, Joilson O. Martins, Maria Regina D’Império Lima, José M. Alvarez, Gustavo P. Amarante-Mendes, Lígia Antunes Gonçalves, Claudio R. F. Marinho, Sabrina Epiphanio

**Affiliations:** 1 Departamento de Análises Clínicas e Toxicológicas, Faculdade de Ciências Farmacêuticas, Universidade de São Paulo, São Paulo, Brazil; 2 Departamento de Imunologia, Instituto de Ciências Biomédicas, Universidade de São Paulo, São Paulo, Brazil; 3 Centro de Ciências Naturais e Humanas, Universidade Federal do ABC, São Paulo, Brazil; 4 Instituto de Investigação em Imunologia, Instituto Nacional de Ciência e Tecnologia (INCT-iii), São Paulo, Brazil; 5 Departamento de Parasitologia, Instituto de Ciências Biomédicas, Universidade de São Paulo, São Paulo, Brazil; 6 Present Address: Center for Global Infectious Disease, Seattle Children’s Research Institute, Seattle, WA USA

**Keywords:** Immunology, Infection

## Abstract

Malaria is an enormous burden on global health that caused 409,000 deaths in 2019. Severe malaria can manifest in the lungs, an illness known as acute respiratory distress syndrome (ARDS). Not much is known about the development of malaria-associated ARDS (MA-ARDS), especially regarding cell death in the lungs. We had previously established a murine model that mimics various human ARDS aspects, such as pulmonary edema, hemorrhages, pleural effusion, and hypoxemia, using DBA/2 mice infected with *Plasmodium berghei* ANKA. Here, we explored the mechanisms and the involvement of apoptosis in this syndrome. We found that apoptosis contributes to the pathogenesis of MA-ARDS, primarily as facilitators of the alveolar-capillary barrier breakdown. The protection of pulmonary endothelium by inhibiting caspase activation could be a promising therapeutic strategy to prevent the pathogenicity of MA-ARDS. Therefore, intervention in the programmed death cell mechanism could help patients not to develop severe malaria.

## Introduction

*Plasmodium* species affecting humans can culminate in severe disease, which includes lung complications as acute respiratory distress syndrome (ARDS) [1–5]. It is known that up to 20 to 30% of severe malaria cases caused by *Plasmodium falciparum* or *Plasmodium vivax* lead to lung injuries. Although not so prevalent, an 80% chance of death is expected in patients who develop malaria-associated ARDS (MA-ARDS) [4, 6, 7].

ARDS pathological findings such as pulmonary edema of non-cardiogenic origin, dyspnea, increased inflammatory mediators, such as cytokines (TNF-α, IL-6, and IL-8) and chemokines/receptors (CXCL-1, CXCL-8, and CXCR4), migration of inflammatory cells to the tissue, and intra-alveolar space, as well as increased capillary alveolar permeability, are key the disease pathogenesis [8–10].

Pulmonary inflammatory infiltrate in MA-ARDS is rich in neutrophils [4] and the reactive oxygen species (ROS) and myeloperoxidase (MPO) released by the neutrophils induce neutrophil extracellular trap (NETs) formation, contributing to the severity of the disease, leading to increased inflammation, tissue damage, and cell death [11].

Different forms of regulated cell death have been implicated in ARDS pathogenesis in human patients in the context of a variety of etiologies and different cell populations [12]. Apoptosis, the most common regulated cell death, can be initiated through the intrinsic pathway, as a consequence of mitochondrial outer membrane permeabilization (MOMP), or via the extrinsic pathway, triggered by death receptors located at the cell surface [13]. Importantly, both pathways were shown to be involved in the acute lung injury induced by sepsis, cystic fibrosis, diffuse alveolar damage (DAD), and hyperoxia [14–18]. Apoptosis extrinsic pathway, initiated by receptors on the cell membrane, such as TRAIL and TNF and their receptors as TNFR-1, and consequently, the adapter molecule FADD induce active caspase-8, which cleaves procaspase-3 to produce active caspase-3, responsible for apoptosis [19–22]. The apoptosis intrinsic pathway is regulated by the mitochondria, when stimulated results in the channeling of pro-apoptotic factors in the cytosol culminating in Caspase-3 activation, DNA fragmentation, proteolytic inactivation of DFFA, and catalytic activity of DFFB. Members of the Bcl-2 protein family such as BAX, BAK, Bad, Bid are also responsible for regulating apoptosis, whereas Xiap is a negative regulator that belongs to the family of apoptosis protein inhibitors, especially at the active site of effector caspase 3 and 7 [19–22].

In ARDS, apoptosis can be modulated by cytokines, chemokines, other inflammatory products, microorganisms, or oxidative stress, which may incite different responses depending on the cell type [14, 23]. Importantly, direct damage to endothelial and epithelial cells is implicated with worsening of the disease [17, 24]. Interestingy enough, the proper elimination of dead cells is essential to the control of ARDS evolution by favoring the disease resolution, whereas increased inflammation and mortality is observed when the phagocytic system fails [25, 26].

Other forms of regulated cell death, such as necroptosis, pyroptosis, NETosis, ferroptosis, and autophagic cell death, were also observed during malaria infection. For instance, our group has previously shown that NETosis occurs in a murine model of malaria-associated ARDS and it contributed to the pathogenesis of the disease [11]. We also demonstrated that autophagy is dysregulated in *P. falciparum* infection [27] and the NLRP3/AIM2–CASP-1 inflammassome is activated in *P. falciparum* and *P. berghei* placental malaria [28].

Both extrinsic and intrinsic pathways of apoptosis are initiated in different cell populations in malaria [29–32]. Neurons and astrocytes from malaria patients may internalize hemozoin, which leads to upregulation of pro-apoptotic proteins and apoptosis of these cells [33]. In MA-ARDS, endothelial cells undergo apoptosis by direct contact with infected red blood cells (iRBC) or its products, such as hemozoin [24, 34]. As in the case of neurons and astrocytes, endothelial cells also upregulate certain apoptosis genes, such as TNF, FAS, Bax, and Bad, suggesting a possible role of these proteins in apoptosis of endothelial cells in MA-ARDS [34]. Finally, it was observed in the lung tissue of *P. falciparum*-infected patients who had edema, an increase in FAS/FASL and caspases-3 and -8 in leukocytes and alveolar cells [35].

Until now, few studies have addressed the apoptotic mechanisms underlying the pathogenesis MA-ARDS [24, 34, 35]. Further studies are needed to understand the types of death and the pathways involved in this process. Thus, this work aimed to investigate the involvement of cell death in the pathogenesis of MA-ARDS in a murine model, by exploring different apoptotic pathways, particularly the caspase signaling.

## Results

### Development of MA-ARDS is associated with increased apoptosis of lung endothelial cells and leukocytes in mice

Apoptosis was evaluated in the lung and bronchoalveolar lavage (BAL) of *P. berghei-*infected mice (ARDS or hyperparasitemia (HP)-developing mice). In our ARDS model, around 50% of mice can die from ARDS, showing pleural effusion, pulmonary hemorrhage, and a different respiratory pattern compared to mice that die with/from hyperparasitemia (HP), which show pale lungs, high amount of infected red blood cells and anemia. According to our predictive model, we were able to identify animals that develop ARDS or HP on the 7th day after infection, as described in the material and methods and previously published [11, 36].

**Fig. 1:**
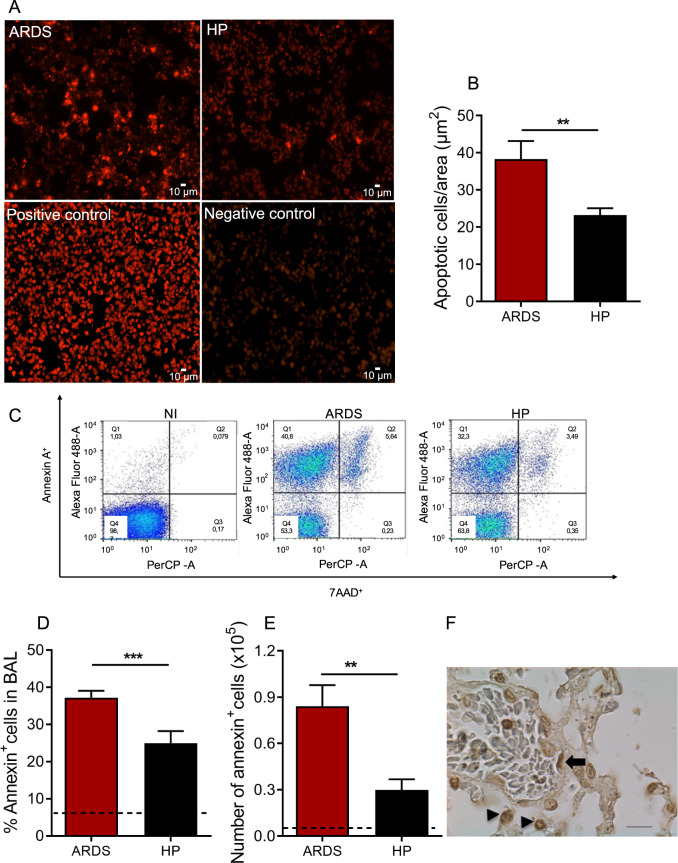
ARDS-developing mice show more abundant apoptotic cells in lungs and bronchoalveolar lavage than HP-developing mice. Lung tissues and bronchoalveolar lavage (BAL) from ARDS- and HP-developing mice were collected from *Plasmodium berghei*-DBA/2 mice infected mice, on the 7th dpi. **A**, **B** Distribution of apoptotic [fluorescent] cells, in the lungs. **B** Quantification of apoptotic cells by pulmonary tissue area (µm^2^). **C**–**E** Apoptotic cells [Annexin V^+^] measured in BAL and necrotic cells stained by 7AAD dye. **C** Representative flow cytometry dot plots from in vivo cell staining (Annexin V^−^ and 7AAD^−^), apoptotic cells (Annexin V^+^), and late apoptotic cells (Annexin V^+^ and 7AAD^+^), performed by flow cytometry. **D** Frequency and **E** number of Annexin V^+^ in BAL. Graphs represent two grouped experiments expressed with mean ± SE, by Mann–Whitney (*n* = 7–9 per group; **p < 0.05; **p < 0.01; ***p < 0.001). Representative image **F** of apoptosis in endothelial cells (arrow) and leukocytes (arrowhead), stained by TUNEL (magnification: ×630, scale bar: 10 μm). Dashed lines: average of NI. 7AAD: 7-Amino-Actinomycin D; BAL: bronchoalveolar lavage; NI: non-infected mice; ARDS: acute respiratory distress syndrome; HP: hyperparasitemia.

The lungs of ARDS-developing mice showed more apoptotic [fluorescent] cells than HP-developing mice (Fig. 1A, B). In addition, ARDS-developing mice showed higher frequency and number of apoptotic cells (total Annexin V^+^) than HP-developing mice (Fig. 1C, D, E) in BAL. Interestingly, there is no significant difference regarding late apoptosis (Annexin V^+^/7AAD^+^) cells in these groups (Fig. 1C). Furthermore, the TUNEL stain allowed us to identify that the apoptotic cells were endothelial cells and leukocytes, particularly alveolar macrophages and neutrophils, showing that these cells are involved in developing malaria-associated ARDS (Fig. 1F).

Our results revealed 32 genes related to cell death pathway were positively or negatively regulated in ARDS and HP groups (Supplementary Fig. 1). After PCR-array, the bioinformatics resources were used to understand which pathways was involved in our model. The KEGG and Biocarta diagrams were generated and transcription of some genes (Supplementary Table 1) was furthter validated by qRT-PCR. TnfrI, Trail, Fadd, RipK1, Casp3, Casp8, Casp9, Dffb, BaK, Bax, Bad, Bid, Xiap, and Akt gene expression was upregulated (Fig. S1A–N), whereas Bcl2 and Bclxl genes (Fig. S1O, P) were downregulated in ARDS-developing as compared to HP-developing mice. These results reinforce the role of apoptosis in the development of MA-ARDS.

### Caspases 3, 6, 8, and 9 signalings are involved in the pathogenesis of MA-ARDS in mice

**Fig. 2:**
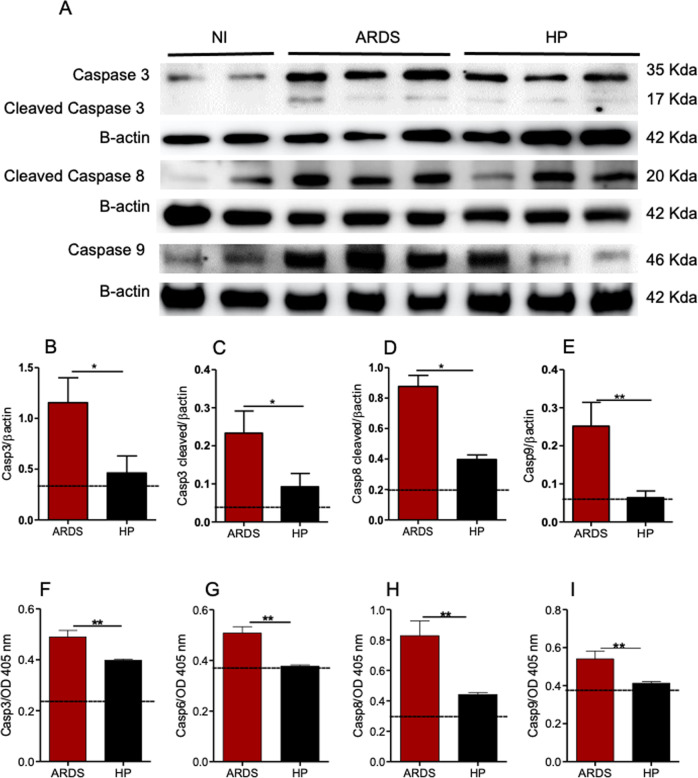
Caspases 3, 6, 8, and 9 signalings are involved in the pathogenesis of ARDS-developing mice. **A**–**E** Protein quantification of caspases 3, 6, 8, and 9 in lung tissue of *Plasmodium berghei*-DBA/2 mice infected related to the β-actin control. **A** Representative inserts of western blot membranes showing the bands of caspases 3, 8, and 9. **F**–**I** Enzymatic activity of caspases quantification. Data analyzed from three grouped experiments expressed with mean ± SE; Mann–Whitney test (NI, *n* = 6; ARDS, *n* = 9; HP, *n* = 9; *p < 0.05 and **p < 0.01). Dashed lines: average of NI. NI: non-infected mice; ARDS: acute respiratory distress syndrome; HP: hyperparasitemia.

Upregulation of caspases 3, 8, and 9 at the mRNA level in ARDS-developing mice may suggest that these regulatory and effector caspases are present in higher amounts and fully available to be activated in these animals on the 7th dpi. To formally test the participation of caspases in the development of ARDS, we performed western-blots and enzymatic activity assays to measure protein expression and activity, respectively (Fig. 2). Indeed, our results showed a significant higher levels of caspase 3 (∼35 Kda), cleaved caspase 3 (∼17 Kda), cleaved caspase 8 (∼20 Kda), and caspase 9 (∼46 Kda), and in the lung of ARDS-developing mice compared to HP-developing mice (Fig. 2A–E). In addition, the enzymatic activity of caspases 3, 6, 8, and 9 was significantly higher in ARDS-developing mice compared to HP-developing mice on the 7th dpi (Fig. 2F–I). In comparison, there was no difference in the activity of caspase 2 in these two groups (data not shown).

### *PbA*-iRBCs induce apoptosis and fluid leakage in PMLEC cultures

**Fig. 3:**
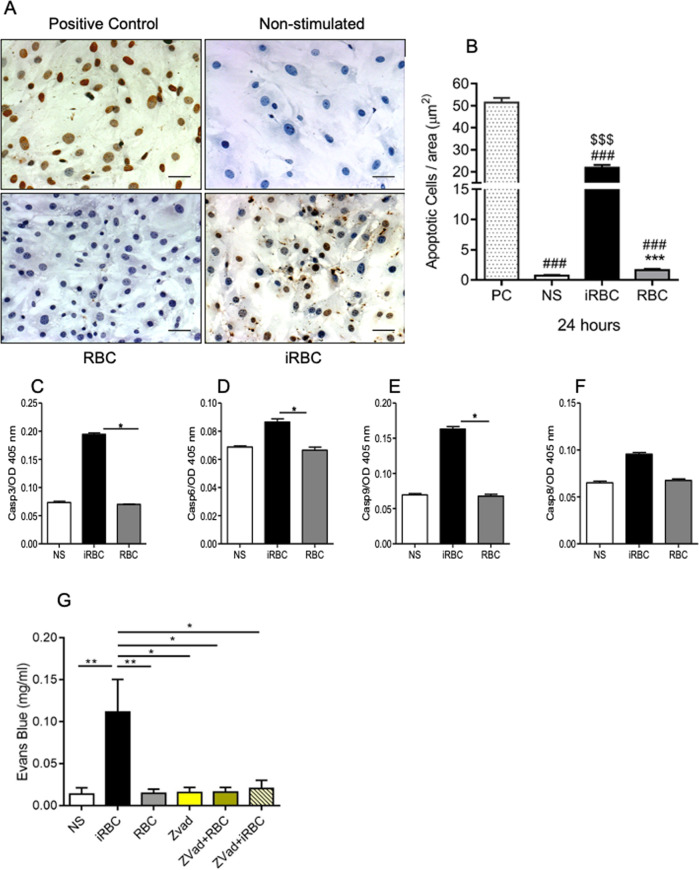
*Plasmodium berghei* ANKA-infected red blood cells activate caspases, induce apoptosis, and promote increased permeability in DBA/2 mice pulmonary endothelial cells. PMLECs from naive DBA/2 mice were stimulated with *Plasmodium berghei* ANKA-infected red blood cells (*Pb*A-iRBCs) or non-infected red blood cells (RBC). **A** Representative images of apoptotic cells (with brown nuclei) stained by TUNEL assay and counterstained with Harris Hematoxylin (blue). **B** Quantification of death cells was analyzed in 15 fields per slide with ×200 magnification. **C**–**F** Enzymatic activity of caspases quantification. Data expressed as mean ± SE; Kruskal–Wallis test, followed by Dunn’s post test, where^###^*p* < 0.001 with respect to the positive control (PC), ^$$$^*p* < 0.001 with respect to NE and ***p < 0.01 with respect to *Pb*A-iRBC. **G** PMLECs were also treated with 50 μM of ZVAD-fmk (caspase inhibitor) for 24 h and compared to NS to evaluate the permeability using Evans blue in a Transwell assay. **B**–**F** Data analyzed from two grouped experiments and expressed as mean ± SE; Kruskal–Wallis test, followed Dunn test (*n* = 7–10 wells/group; *p < 0.05, **p < 0.01, and ***p < 0.001). **G** ANOVA, followed Tukey multiple comparison test. NS: non-stimulated; iRBC: *Plasmodium berghei* ANKA-infected red blood cells; RBC: non-infected red blood cells; Zvad: ZVAD-fmk (caspase inhibitor) treatment.

TUNEL staining revealed ten times more apoptotic PLMECs after exposure to *Pb*A-iRBC compared to non-infected RBCs (Fig. 3A, B). In addition, upon exposure to *Pb*A-iRBC, the PLMECs presented a significant increase of caspases 3, 6, and 9 activity (Fig. 3C–E), and no difference was found for caspase 8 (Fig. 3F). Furthermore, when PLMECs seeded over a transwell membrane system were treated with the pan-caspase inhibitor ZVAD-fmk, a significant reduction in Evans Blue leakage was observed (Fig. 3G). These data indicate that *Pb*A-iRBCs promote apoptosis of endothelial cells and, consequently, increased of vascular permeability.

### Caspase inhibition prevents the development of MA-ARDS in mice

**Fig. 4:**
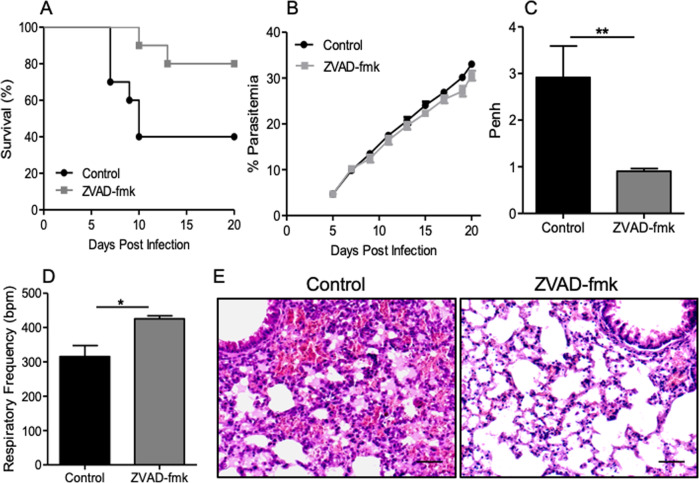
Inhibition of caspases prevents ARDS in *P. berghei* ANKA-infected DBA/2 mice. Infected mice were treated with ZVA-fmk (5 mg/kg) with a single dose on the 3rd dpi. **A** Survival curve analyzed by the Log-rank test, where *p* < 0.05 (*n* = 20 mice); **B** Parasitemia throughout the infection; **C** increased respiratory pause (Penh), and **D** respiratory rate on the 7th dpi. Data representative of two independent experiments expressed with mean ± SEM by Mann–Whitney test (*n* = 3 NI; *n* = 10 control-infected mice; *n* = 10 infected and ZVAD-fmk-treated mice; **p < 0.01). ARDS: acute respiratory distress syndrome; HP: hyperparasitemia. **E** Photomicrographs of ARDS-developing mice lungs that died on the 10th day after infection (untreated controls) and ZVAD-fmk-treated. Note hemorrhagic and edematous areas in the control group (ARDS-developing mice); ×400 magnification; Scale bar 50 μm.

To investigate whether MA-ARDS could be prevented by inhibiting caspase activation in vivo*, P. berghei* ANKA-infected DBA/2 mice were treated or not with a single dose of ZVAD-fmk (a pan-caspase inhibitor) on the 3rd dpi and analyzed until the 20th dpi. In the control group, 60–70% of mice died by ARDS between 7th and 12th dpi, while 80–90% of ZVAD-fmk-treated mice survived, although no significant difference in parasitemia was observed (Fig. 4A, B). The respiratory capacity of ZVAD-fmk-treated mice was recovered, as Penh decreased and RF increased (Fig. 4C, D). In addition, histological analysis of the lungs on the 10th dpi revealed that ZVAD-fmk treated mice had reduced inflammatory infiltrate and little or no edema. In contrast, control mice showed severe hemorrhage, edema, and inflammatory infiltrate (Fig. 4E). These results support the role of caspases in the pathogenesis of MA-ARDS in mice.

## Discussion

In the present study, we found that apoptosis contributes to the pathogenesis of malaria-associated ARDS, primarily as facilitators of the alveolar-capillary barrier breakdown.

The alveolar epithelium is considered one of the main components that act in the alveolar space barrier. Importantly, the alveolo-capillary barrier is impaired during ARDS, leading to increased vascular permeability and compromising the liquid clearance capacity of the alveolar space [13, 37, 38].

Apoptosis, a highly programmed cell death process, is associated with caspase-dependent characteristic morphological and biochemical modifications and plays an important role in the genesis of several physiological and pathological processes [20, 38], including the development of malaria-associated ARDS [35].

Using a murine model of *Pb*A-infected DBA/2 mice previously established by our research group [36], we show that ARDS-developing mice present a higher number of apoptotic cells in the lungs and bronchoalveolar lavage when compared to HP-developing mice. Our results suggest that apoptosis of accumulated inflammatory cells and/or endothelial cells contributes to the pathogenesis of the disease. The increase in the number of neutrophils in the lungs and BALs of infected mice is associated with the release of myeloperoxidase and reactive oxidative species (ROS) as well as the formation of neutrophils extracellular trap (NET) [11], all implicated with the induction of apoptosis in different cell types [39–41]. Interestingly, apoptosis of endothelial cells can be triggered by any of these inflammatory mediators, by direct contact with *Plasmodium* iRBCs or by the hemozoin secreted by the parasite [24, 34].

Our results showed that the pro-apoptotic genes Tnfr-1, Trail, Fadd, Ripk-1, Casp-3, Casp-8, Casp-9, Dffb, Bak, Bax, Bad, Bid, Xiap are positively regulated in ARDS-developing mice compared to HP-developing mice, suggesting that both extrinsic and intrinsic pathway of apoptosis may participate in the pathogenesis of malaria-associated ARDS. Interestingly, Lee et al. have observed higher concentrations of TRAIL in the bronchoalveolar lavage fluid of ARDS patients [42]. In contrast, Albertine et al. demonstrated an increase in BAX in the lung tissue of patients who died with ARDS or ALI [43], and BAX induction in diffuse alveolar damage might increase the susceptibility of alveolar epithelial cells to apoptosis [16]. We quantified caspases-2, -3, -6, -8, and -9 in lung tissue and observed that caspases-3, -8, and -9 showed a significant difference in ARDS-developing compared to HP-developing mice. As caspases-8 and -9 activate the effector caspase-3, our data support that both extrinsic and intrinsic pathways may operate in the pathogenesis of ARDS-developing mice. Interestingly, it has been observed in the lungs of patients with severe malaria with pulmonary edema a significant increase in caspases-3 and -8 [35]. Semi-quantitative immunohistochemistry assays demonstrated a prevalence of caspase-3 in cells of the alveolar wall in post-mortem samples from patients with ARDS, reinforcing the participation of the apoptotic cascade activation in the epithelial cells that line the alveolar air spaces [43].

We also tested whether *Pb*A-iRBCs could induce apoptosis in PMLECs of naive DBA/2 mice. Our data revealed the activation of effector caspases-3 and -6 and the initiator caspase-9 and an associated increased in permeability of PLMEC cultures, as measured by extravasation of Evans Blue. Importantly, the permeability of *Pb*A-iRBCs-treated PLMECs was completely abolished by the pan-caspase inhibitor ZVAD-fmk, suggesting that *Pb*A-iRBCs can direct induce apoptosis in endothelial cells, thereby increasing vascular permeability. Pulmonary endothelial cell death was also demonstrated to be inhibited by ZVAD-fmk by others [44].

Finally, to confirm the involvement of apoptotic caspases in the pathogenesis of MA-ARDS, we treated *Pb*A-infected mice with ZVAD-fmk.

Mingdong Liu et al., using a rat model of severe acute pancreatitis, showed 0.3 ml zVAD-fmk (50 mmol/L, injected by intraperitoneally) inhibits pulmonary cell apoptosis, suppresses the sequestration of neutrophils, downregulates inflammatory cytokines, and alleviates lung injury, analyzed in different time points, and the last one was 24 h [45]. Other authors used different lung models, a single dose of zVAD-fmk concentration, and administration drug via also observing the decreased apoptotic process and endothelial protective mechanism [46, 47].

Our results clearly showed a positive impact of in vivo caspase inhibition on the survival outcome. Around 80% of mice treated with ZVAD-fmk survived compared to 40% of non-treated mice, despite the similar levels of parasitemia observed in both groups. Furthermore, the Pehn was higher, and the respiratory rate was lower in the control animals compared to the ZVAD-fmk-treated group. In addition, hemorrhagic and edematous areas were observed in lungs of the control group, as opposed to the ZVAD-fmk-treated mice.

Taken together, our results shown that inhibition of caspases prevents all symptoms associated with MA-ARDS and significantly ameliorate survival.

In conclusion, the present study indicates that in murine malaria-associated ARDS, lung caspases are activated, promoting apoptosis of endothelial cells, which has a causal link with the pathogenesis of MA-ARDS. Notably, intervention in apoptosis pathway through the inhibition of caspases protects the alveolar-capillary barrier in pre-clinical trials to MA-ARDS. Therefore, these results indicate that apoptosis interference may be considered as a therapeutic intervention to prevent patients with malaria from developing ARDS and, consequently, reducing significantly the mortality rate from this disease.

## Materials and methods

### Mice, parasites, and euthanasia

Male DBA/2 mice with 6–10 weeks old (purchased from the Department of Parasitology, University of São Paulo, Brazil) were infected by intraperitonial injection (IP) with 1 × 10^6^ *P. berghei* ANKA (*Pb*A) (clone 1.49L)-infected red blood cells (iRBC), as previously defined [31]. Mortality and parasitemia were monitored daily, being the latter determined by Giemsa staining followed by microscopic counting and expressed as a percentage (counted iRBCs per non-infected RBCs). Mice were euthanized by i.p. infection of ketamine (150 mg/kg) and xylazine (15 mg/kg) on the 7th day post-infection (dpi) or when signs of suffering or imminent death were observed. No blinding methods were used in the reported experiments.

### Experimental outline

DBA/2 mice were infected with 10^6^ *Pb*A-iRBCs and classified as ARDS-developing or hyperparasitemia (HP)-developing mice before death as previously described [48]. Briefly, we used two groups of infected mice: the survival group (infected control) and the euthanized group, in which the mice were euthanized on the 7th dpi (10–12 mice per group). By using enhanced pause (Penh), respiratory frequency (RF), and when either sign of suffering or imminent death were observed parasitemia as predictive criteria, we established cut-off values using receiver operating characteristic (ROC) curves for these parameters measured on the 7th dpi based on data from mice whose cause of death was known. In the survival group, mice presenting red congested lungs and pleural effusion or at necropsy, the cause of death was nominated as ARDS. Mice without pleural effusion that died after 13th dpi showing pa lungs and high levels of parasitemia, the cause of death was designated as hyperparasitemia (HP). Subsequently, we retrospectively diagnosed the euthanized mice as suffering from ARDS or HP by comparing their respiratory patterns and parasitemia measured on the 7th dpi with the cut-off values from the survival group at the end of each experiment (20th dpi) [36, 48]. Pulmonary tissues were used to screen for apoptotic gene expression by PCR array, identification of cell death by TUNEL assay, and protein quantification by western blot from the euthanized animals on the 7th dpi.

### Determination of respiratory pattern

RF and Penh were measured on the 7th dpi by placing the mice in an unrestrained whole-body plethysmography chamber (Buxco Electronics, USA) for 10 min (basal level), as formerly described [48].

### Histology

The mice lungs were collected on the day of death and placed in 10% buffered formalin solution and, after 24 h, transferred to 70% ethanol. Lungs were then embedded in paraffin, sliced in 5 μm thickness sections and stained with Hematoxylin and Eosin dye.

### Apoptosis detection assays

On the 7th dpi, the lungs of the euthanized animals were perfused with 10 ml of PBS (1X) and then dipped in 2-methylbutane (Sigma-Aldrich) for 5 min. Subsequently, the lungs were involved in Tissue-Tek O.C.T.© (Sakura Fineteck, CA, USA), frozen in liquid nitrogen, cut in a 7 μm thickness, and deposited on pre-silanized histological slides (γ-Methacryl-Oxipropyl-Methoxysilane, Sigma-Aldrich). The frozen lung sections were fixed in 4% paraformaldehyde for 20 min and permeabilized with 0.1% Triton-X in 0.1% Sodium Citrate solution. The TUNEL reaction was performed with In Situ Cell Death Detection Kit Fluorescein (Roche Diagnostics, Mannheim, Germany), protected from light, in a humid chamber at 37 °C for 60 min. Apoptotic cells were observed in the Zeiss fluorescence microscope (Axio Imager M2) and counted using Image-Pro Plus software version 6.0 (Cybernetics Medium). The TUNEL Assay in paraffin-embedded tissue sections was performed to recognize the form of apoptotic nuclei, using In Situ Cell Death Detection Kit, POD Roche, cat.#11684817910 (Roche Diagnostics, Mannheim, Germany).

### Annexin V stanning in bronchoalveolar lavage

Detection of apoptotic cells in bronchoalveolar lavage was performed by phosphatidylserine (PS) with annexin-V staining using Annexin-V-FLUOS Staining Kit (cat.#11988549 001; Roche Diagnostics, Mannheim, Germany). Cells were counted and 10^5^ to 10^6^ cells were washed with PBS 1x and centrifuged at (1200 rpm for 5 min). The cells were incubated with Annexin-V-FLUOS and 7AAD (Amino-Actinomycin D; BD Biosciences, USA, cat.# 54-38981E) for 10 min. After, the cells were filtered and went through on the FACScalibur or FACScantoII cytometer (BD Biosciences, USA) with Hepes buffer.

### Screening of apoptosis-related gene expression

The mRNA from lung tissues collected on the 7th dpi was extracted with the RNeasy Mini Kit (Qiagen) and cDNA synthesis reaction was performed with the HT kit First Strand cDNA (Qiagen). The extracted CDNA + SYBR Green PCR Mastermix (Applied Biosystems) was added to the 96-well plates of the Apoptosis RT2 Profiler PCR Array (PAMM-012ZC, Qiagen). Each PCR-Array plate comprises probes for 82 programmed cell death genes and 4 constitutive genes, as controls of the reaction, used to analyze standardization. The results were analyzed by the relative quantification (2^−ΔΔCT^) method.

### Gene ontology and bioinformatics analysis

The results of apoptosis PCR-Array were analyzed by gene ontology. Genes showing statistically significant differences (*p* ≤ 0.1) differences between ARDS and HP were evaluated in the DAVID bioinformatics program (David Bioinformatics Resources 6.7) [49]. The selected apoptosis genes were analyzed by molecular interaction diagrams employing KEGG and Biocarta models. These models, crossing references with many databases, generated an autonomous system to link the genome to molecular levels, showing the pathways of cellular functions [50, 51].

### Quantitative reverse transcription-polymerase chain reaction (qRT-PCR)

In order to validate the results obtained in the PCR-array, other samples of mRNA pulmonary tissue were evaluated by qRT-PCR for genes that presented statistical significance on the 7th dpi. The mRNA extraction was performed using the RNEasy Mini Kit (Qiagen), following the manufacturer protocol. The mRNA quality and concentration were verified in the Nanodrop 2000 (Thermo Scientific). The cDNA synthesis was performed with 1 μg of RNA for each sample using the First Strand cDNA Synthesis kit for RT-PCR (Roche). The qRT-PCR was performed using SYBR Green PCR Mastermix (Applied Biosystems). The relative gene expression results were obtained through the 2^−ΔΔCT^ method. All samples were normalized to the expression of the constitutive gene, HPRT (Hypoxanthine-guanine phosphoribosyltransferase), and uninfected control. The gene expression was expressed as fold increase in relation to the non-infected mice. The primers used to validate anti- and pro-apoptotic genes are shown in Supplementary Table 1 (Table S1).

### Extraction and quantification of pulmonary tissue proteins

Pulmonary tissues were collected on the 7th dpi, placed in a Ripa buffer supplemented with protease and phosphatase inhibitor (Abcam 5872S), and maintained at 4 °C. The tissue was homogenized with 5 mm stainless steel beads (Qiagen) on Tissue Lyser equipment (Qiagen) and then centrifuged at 13,000 rpm for 20 min. The supernatant was weighed and frozen at −80 °C. The Pierce BCA Protein Assay Kit (Thermo Scientific) was used to quantify the proteins.

### Western blot

Fifty micrograms of proteins from lung tissues were placed in loading buffer (Biorad), denatured at 99 °C, and run in 15% Tris-glycine SDS polyacrylamide gel, according to the manufacturer protocol (Biorad). The gels were transferred to PVDF membranes 0.45 μm (Immobilon - Millipore) by electrophoresis for 90 min at 100 V. The membranes were blocked in 5% skimmed milk diluted in TBS-T (Tris Buffered Saline with Tween) for 1 h. Membranes were incubated with primary antibodies [anti-caspase 3 (caspase-3 p11 (K-19) sc-1224; Santa Cruz); anti-caspase cleaved 3 (ab13847; Abcam; 1:500) anti-caspase 8 (ab25901; Abcam; 1:1000), anti-caspase 9 (ab28131; Abcam; 1:1000)] diluted in TBS-T buffer, for 12–14 h under gentle shaking at 4 °C and washed 5 times with TBS-T buffer. Anti-Rabbit IgG-HRP (Sigma Aldrich-A0545; 1:10,000) or anti-Mouse IgG-HRP (Sigma Aldrich-A9917; 1:50,000) secondary antibodies was used and kept for 1 h at room temperature (RT). The membranes were then washed 5 times for 5 min with TBS-T buffer and revealed by Amersham ECL Prime Western Blotting Detection Reagent (GE Healthcare) on the Amersham Imager 600-GE equipment. Once again the membranes were washed and blocked with 5% skimmed milk for 2 h at RT under gentle agitation, incubated with monoclonal anti-β-actin peroxidase Mouse antibody (Sigma-Aldrich-A3854; 1:50,000) and revalued with ECL. The band densitometry was performed by Image Studio Lite software, and the graphs were generated using the GraphPad Prism 5.0 software.

### Isolation of pulmonary endothelial cells

Primary Microvascular lung endothelial cells (PMLEC) from DBA/2 mice were isolated, as described before [52] and used after 4 passages. Briefly, all blood was withdrawn from euthanized mice by cutting the carotid arteries and pulmonary tissues were cut into 1 mm^3^ fragments and added to 6-well culture plate with low-glucose DMEM medium supplemented with 20% FBS and 1x antibiotic-antimycotic (ThermoFisher Scientific). The tissues were incubated at 37 °C with 5% CO_2_ and removed after 60 h. For cell cultured and expansion, complete DMEM medium was changed every 2–3 days and frequently observed for endothelial cell purity and morphology.

### Isolation and synchronization of infected red blood cells

In order to obtain mature trophozoites and schizonts, iRBCs were synchronized, as previously described [53]. *Pb*A-iRBC, obtained from the blood of infected DBA/2 mice, were added in a culture bottle containing RPMI medium with 20% fetal bovine serum. Then, the gas mixture containing 85% N_2_, 10% O_2_, and 5% CO_2_ (White Martins) was added and incubated at 37 °C for 14 h. After, schizonts were isolated using magnetic separation column MACS® (CS columns 25 -Miltenyi Biotec, Bergisch Gladbach, Germany). The magnetic column attached to the magnet [54] was first stabilized with 40 ml of unsupplemented RPMI medium and, subsequently, the suspension of synchronized-iRBCs was added. At this stage, the non-infected RBCs (RBCs), passing freely through the column, were collected. The late mature forms (schizonts and mature trophozoites) were attracted by the magnet and retained in the column. Next, these iRBCs were washed with RPMI, resuspended in DMEM medium, and counted for the next assays.

### Caspase activity assay

Proteins extracted from the lungs of non-infected or *P. berghei* ANKA-infected DBA/2 mice, and also from cultures of PMLECs from DBA/2 mice (PLMEC) [stimulated or non-stimulated with iRBC or red blood cells (RBC)] were used. The ApoTarget kit (Thermo Scientific) was employed to quantify the aminoacid sequences VDVAD (caspase 2), DEVD (caspase 3), VEID (caspase 6), IETD (caspase 8), and LEHD (caspase 9).

### In vitro pulmonary endothelial cell stimulation for detection of apoptosis

Isolated PMLECs were co-cultured with 25 infected *Pb*A-iRBC or 25 RBCs (25 per endothelial cell) for 24 h. Apoptosis was detected by TUNEL staining, anti- and pro-apoptotic genes were analyzed by qRT-PCR, and cytokines were measured in the supernatant from these cells by ELISA assays.

### TUNEL reaction in PMLEC

The TUNEL assays were performed in vitro using 24-well plates, where we placed 5 × 10^5^ PLMEC per well. The wells containing the adhered PLMECs were stimulated by either RBC or *Pb*A-iRBC schizonts for 24 h (25 per PLMEC). After this period, the cells were washed and fixed with 1% paraformaldehyde and postfixed with 70% ethyl alcohol to 30% acetic acid. The TUNEL assay in adherent cells was performed using the Apoptag Plus Peroxidase kit in situ (EMD Millipore - USA). After this test, the coverslips were mounted on conventional slides using the Tissue Tek Paraffin Wax medium.

### Measure of PMLEC permeability in vitro

To analyze the lung vascular permeability, PLMECs were plated on permeable membrane inserts with 0.4 μM pores (Transwell Corning) pre-treated with gelatin 0.2% in 1 × PBS (gelatin from bovine skin, G9391, Sigma-Aldrich) and then coupled in 24-well polystyrene plates at a concentration of 2.2 × 10^4^ cells per insert and maintained in DMEM culture at 37 °C, as previously described [33]. After 96 h, once the cells reached confluency, inhibitor of caspases zenzyloxycarbonyl–Val–Ala–Asp–fluoromethylketone (ZVAD-fmk, ALX-260-020-M00; 02051804; ENZO Life Sciences; New York, USA) was used. After 2 h of incubation with 50 μM of ZVAD-fmk or just 20% FBS-supplemented DMEM culture medium, *Pb*A-iRBC or RBC (25 per PLMEC) were added for 1 h. Hank’s balanced salt solution subsequently replaced the culture medium in the lower compartment. In the upper compartment of each insert in contact with the cells, 200 μl of Evans Blue was incubated at a 2 mg/ml concentration at 37 °C. The liquid from the lower compartment was collected after 30 min and analyzed in a spectrophotometer at a wavelength of 650 nm (NanoDrop 2000, Thermo Fisher Scientific). Evans Blue concentration was measured from a standard curve (0.2–0.0031 mg/ml) as previously described [36, 55].

### In vivo caspase inhibition assay

*P. berghei* ANKA-infected DBA/2 mice were treated with an apoptosis inhibitor, ZVAD-fmk (fluoro-methylketone - Invivogen). The animals received a single dose of ZVAD-fmk (5 mg/kg) or DMSO (control), i.p., on the 3rd dpi, and monitored daily for parasitemia, for 20 days. The survival, parasitemia, respiratory capacity (Penh and FR), and pathological findings were analyzed.

### Statistical analysis

Statistical analyzes were performed using the GraphPad Prism 5.0 software for analysis and graphing. Data were analyzed for normality by Kolmogorov-Smirnov or Shapiro Wilk tests and Bartlett’s test for variance. Non-parametric variables were compared using Mann–Whitney test between ARDS and HP groups. Group variance was tested using Bartlett’s test for homogeneity of variances (homoscedasticity). When this and other assumption (normal distribution and data independency) were met, one-way ANOVA was used to test for differences between multiple groups. For analysis of three groups or more, Kruskal–Wallis test was used followed by Dunn’s post hoc test. The one-way ANOVA with Tukey multiple comparison test were used for parametric variables. For the survival curves, Log-rank test was applied. The differences between the groups were considered significant when *p* ≤ 0.05 (5%). To establish data cut-off, ROC curves were generated using the results of the control group in MedCalc version 8.2.1.0. No statistical methods were used to estimate sample size.

## Supplementary information

Supplementary Figure 1

Legends -Supplementary Figure 1

Table - Supplementary 1

aj-checklist

## Data Availability

The datasets used and/or analyzed during the current study are available from the corresponding author on reasonable request.
