# Supplementary figures and images for: Blockade of caspase cascade overcomes malaria-associated acute respiratory distress syndrome in mice
Source: Cell Death Dis. 2022 Feb 10;13(2):144. doi: 10.1038/s41419-022-04582-6 (PMC8831525; doi:10.1038/s41419-022-04582-6)

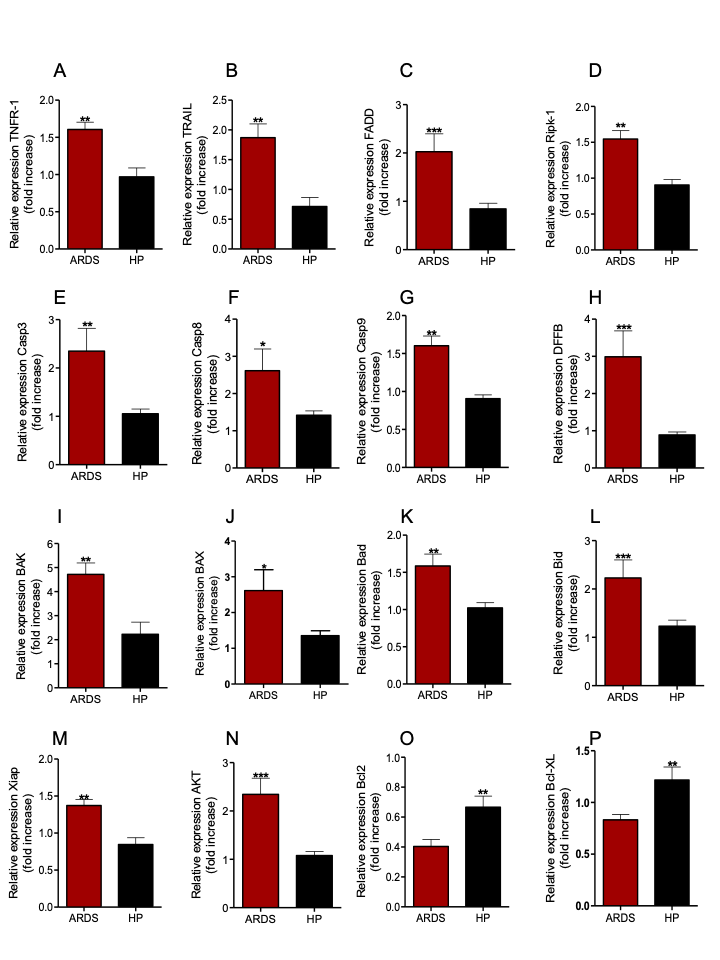

Supplement: Supplementary file 1 — Supplementary Figure 1 [file 41419_2022_4582_MOESM1_ESM.tif]
