# Supplementary material for: Blockade of caspase cascade overcomes malaria-associated acute respiratory distress syndrome in mice
Source: Cell Death Dis. 2022 Feb 10;13(2):144. doi: 10.1038/s41419-022-04582-6 (PMC8831525; doi:10.1038/s41419-022-04582-6)
Supplement: Supplementary file 2 — Legends -Supplementary Figure 1 [file 41419_2022_4582_MOESM2_ESM.docx]

**Fig. S1:** **Pro-apoptotic genes are up-regulated in ARDS-developing mice compared to HP-developing mice.** Relative expression of pro-apoptotic (A-N) and anti-apoptotic genes (O and P) in lungs of *Plasmodium berghei*-DBA/2 mice infected mice, on the 7^th^dpi. Graphs represent two grouped experiments expressed with mean ± SE by Mann-Whitney test (ARDS, n=12 and HP, n=9; *p < 0.05, **p < 0.01 and ***p < 0.001). ARDS: acute respiratory distress syndrome; HP: hyperparasitemia.
