# Supplementary material for: Blockade of caspase cascade overcomes malaria-associated acute respiratory distress syndrome in mice
Source: Cell Death Dis. 2022 Feb 10;13(2):144. doi: 10.1038/s41419-022-04582-6 (PMC8831525; doi:10.1038/s41419-022-04582-6)
Supplement: Supplementary file 3 — Table - Supplementary 1 [file 41419_2022_4582_MOESM3_ESM.docx]

**Table S1 – List of primers containing pro and anti-apoptotic genes and constitutive gene.**

| Gene | Product  Amplified | Genbank number | Sense | Antisense |
| --- | --- | --- | --- | --- |
| AKT | 180 | NM_009652.3 | 5’gggaaggtgattctggtgaa-3’ | 3’-gtcgtgggtctggaatgagt-5’ |
| Bad | 187 | NM_007522.3 | 5’-aggacttatcagccgaagca-3’ | 3’-gctcaaactctgggatctgg-5’ |
| Bak | 115 | NM_007523.2 | 5’-tggaacccaacagcatctt-3’ | 3’-tgtgggctgaagctgttcta-5’ |
| Bax | 173 | NM_007527 | 5’-tgcagaggatgattgctgac-3’ | 3’-gatcagctcgggcactttag-5’ |
| Bcl2 | 212 | NM_009741 | 5’-tgcctacaagaaagcctggt-3’ | 3’-aaaatgcttctcggcacaat-5’ |
| Bcl-XL | 150 | [XM_006498611.5](https://www.ncbi.nlm.nih.gov/entrez/viewer.fcgi?db=nucleotide&id=1907134614) | 5’-gctgggacacttttgtggat-3’ | 3’tgtctggtcacttccgactg-5’ |
| Bid | 206 | NM_007544.3 | 5’-ctctgcgttcagcttgagtg-3’ | 3’-cagaagcccacctacatggt-5’ |
| Casp3 | 205 | NM_001284409.1 | 5’-cgtgggaaagtgaaccagat-3’ | 3’-tgctttccaagtcctgtgtc-5’ |
| Casp8 | 175 | NM_009812.2 | 5’-tctatggaacggatgggaag-3’ | 3’-gtgtggttctgttgctcgaa-5’ |
| Casp9 | 176 | [NM_001355176.1](https://www.ncbi.nlm.nih.gov/entrez/viewer.fcgi?db=nucleotide&id=1243938451) | 5’-ggcccttccttcctctcttcatct-3’ | 3’-tctctgctcctttgctgtga-5’ |
| DFFB | 153 | NM_007859 | 5’-gctcaaatcggtgcagtaca-3’ | 3’-ctgttgccataggggttgat-5’ |
| FADD | 162 | NM_010175.5 | 5’-gcctcaagcctaccatgttc-3’ | 3’-agtctggggagtcaagagca-5’ |
| HPRT | 186 | NM_013556 | 5’aagcttgctggtgaaaagga-3’ | 3’-ttgcgctcatcttaggcttt-5’ |
| RIPK1 | 197 | NM_009068.3 | 5’-ccctgtgcccaataaactgt-3’ | 3’-gtgtaggcggcttcagtctt-5’ |
| TNFR-1 | 217 | [NM_011609.4](https://www.ncbi.nlm.nih.gov/entrez/viewer.fcgi?db=nucleotide&id=158533987) | 5’-ctgtatgctgtggtggatgg-3 | 3’-ccaggttcatcttggaaagc-5’ |
| TRAIL | 236 | NM_009425.2 | 5’-atcggaaaggctgtgagaaa-3’ | 3’-agtgatgacttggggaccag-5’ |
| XIAP | 216 | NM_009688.2 | 5’-catggagctgatgtggctaa-3’ | 3’-gagccggttattctgtgacc-5’ |
